# Smart Bandaid Integrated with Fully Textile OECT for Uric Acid Real-Time Monitoring in Wound Exudate

**DOI:** 10.1021/acssensors.2c02728

**Published:** 2023-03-17

**Authors:** Danilo Arcangeli, Isacco Gualandi, Federica Mariani, Marta Tessarolo, Francesca Ceccardi, Francesco Decataldo, Federico Melandri, Domenica Tonelli, Beatrice Fraboni, Erika Scavetta

**Affiliations:** † Department of Industrial Chemistry “Toso Montanari”, University of Bologna, Viale Risorgimento 4, 40136 Bologna, Italy; ‡ Department of Physics and Astronomy “Augusto Righi”, University of Bologna, Viale Berti Pichat 6/2, 40127 Bologna, Italy; § Plastod S.p.A., Via Walter Masetti 7, Calderara di Reno, 40012 Bologna, Italy

**Keywords:** OECT, organic electrochemical transistor, sensor, PEDOT:PSS, textile, wearable, bioelectronics, wound healing, uric acid sensing, non-enzymatic, smart dressing

## Abstract

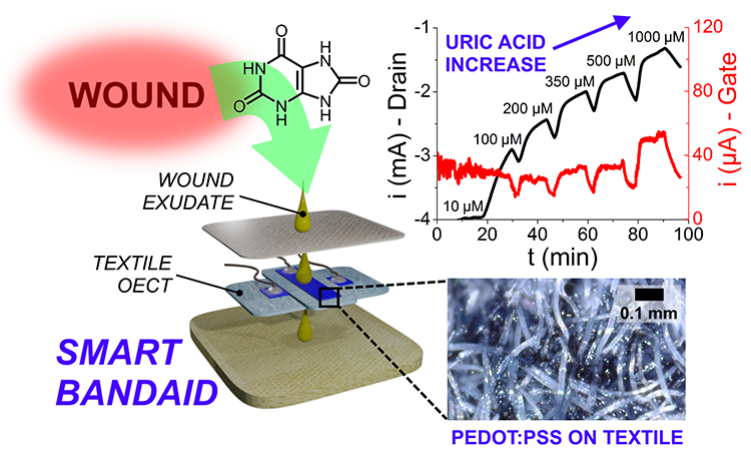

Hard-to-heal wounds (i.e., severe and/or chronic) are typically associated with particular pathologies or afflictions such as diabetes, immunodeficiencies, compression traumas in bedridden people, skin grafts, or third-degree burns. In this situation, it is critical to constantly monitor the healing stages and the overall wound conditions to allow for better-targeted therapies and faster patient recovery. At the moment, this operation is performed by removing the bandages and visually inspecting the wound, putting the patient at risk of infection and disturbing the healing stages. Recently, new devices have been developed to address these issues by monitoring important biomarkers related to the wound health status, such as pH, moisture, etc. In this contribution, we present a novel textile chemical sensor exploiting an organic electrochemical transistor (OECT) configuration based on poly(3,4-ethylenedioxythiophene):polystyrene sulfonate (PEDOT:PSS) for uric acid (UA)-selective monitoring in wound exudate. The combination of special medical-grade textile materials provides a passive sampling system that enables the real-time and non-invasive analysis of wound fluid: UA was detected as a benchmark analyte to monitor the health status of wounds since it represents a relevant biomarker associated with infections or necrotization processes in human tissues. The sensors proved to reliably and reversibly detect UA concentration in synthetic wound exudate in the biologically relevant range of 220–750 μM, operating in flow conditions for better mimicking the real wound bed. This forerunner device paves the way for smart bandages integrated with real-time monitoring OECT-based sensors for wound-healing evaluation.

Wound care is a commodity sector constantly on the rise as new modern medical wound care materials are being studied, such as hydrogels,^1−3^ antibacterial nanofibers,^4−6^ or metal nanoparticle-based dressings,^7−9^ which aim to aid in wound-healing phases, mainly following a preventive approach against eventual worsening of wound conditions. A more active outlook toward wound care can be found in the most recent development in the field of smart bioadhesives,^10^ a broad category of hydrogel-based materials whose main purpose is to seal the wounded area while still being capable of resisting mechanical deformations.^11,12^ Bioadhesives can be either natural (featuring biopolymers such as chitosan, alginate,^13^ or fibroin^14^) or synthetic (cyanoacrylates^15^ or dual-network polymers^16^); the latter ones present interesting tunable physical–chemical properties like adhesion strength, elasticity, and stiffness, drug delivery capabilities,^17^ self-healing abilities,^18^ and immunomodulation^19^ that can be achieved through hydrogel side chain functionalization^20^ or cross-linking.^21^ However, to date, few to no commercial devices exist to improve the medical personnel’s ability to effectively monitor the wound-healing status without removing the bandages and visually inspecting the affected area. This operation is particularly critical for patient’s health, as any unwanted disturbances of the wound bed could lead to pain, stress, physical damage, or infection. Especially for severe and/or chronic wounds such as those deriving from third-degree burns, skin grafts, diabetes-related complications, or compression injuries in bedridden patients, the risk of infection is a major concern. The rise of the point-of-care (PoC) analysis approach in the biomedical diagnostic field^22,23^ and its synergy with the Internet-of-Things (IoT) principles, especially in the wake of the COVID-19 pandemic—as it became a necessity to decentralize the workload of the medical facilities^24^—is pushing the research in the wound care sector toward the development of novel systems for the detection of useful chemical and physical parameters strongly correlated with the wound health status, such as pH,^25^ moisture,^26^ calcium ion,^27^ and temperature.^28^ A real-time, quantitative, and continuous analysis of these biometrics could give the medical personnel—or the patient itself—the ability to control the wound-healing status without disturbing the affected area, thus improving the patient’s recovery through better-targeted therapies, leading to cost reductions for the healthcare system and optimization of the treatment quality. In this regard, bioadhesives and other types of wound dressings can be made “smart” by embedding suitable sensors in their matrix, combining the wound treatment and protection properties provided by the medication with the retrieval of valuable biomedical data, possibly leading to the development of a closed-loop system providing on-demand delivery of specific drugs regulated by the real-time wound health monitoring.^29,30^ An additional improvement is the introduction of wireless systems for device powering and data transfer (such as Bluetooth, Wi-Fi, or NFC),^31,32^ allowing a good implementation of the PoC/IoT principles while greatly reducing the medication encumbrance on the patient and the medical personnel, as these devices need to be portable and compact. The main challenges in the development of smart dressings lie in the production process itself, as it needs to grant a functional integration between the bioadhesive and the sensor while being cost-effective for commercialization purposes. The most recent techniques involved in the production of bioadhesives with a well-defined morphology and homogenous drug-loading distribution are, for example, three-dimensional (3D)-printing^33,34^ and electrospinning,^35,36^ while low-cost and easy-to-scale processes concerning the sensor assembly are ink-jet printing, screen printing, laser cutting, or scribing.^30,37−40^ Among the various biomarkers associated with the different wound-healing phases, uric acid (UA) is one of the most interesting. In healthy conditions, uric acid in wound exudate ranges between 220 and 750 μM, as determined^41^ by Trengove et al. Deviations from this normal biological interval are associated with a radically different wound status. A UA concentration lower than 220 μM can be associated with the presence of pathological bacterial strains in the wound,^42^ such as *Staphylococcus aureus*, *Pseudomonas aeruginosa*, *Proteus mirabilis*, *Escherichia coli*, and Corynebacterium spp. of exogenous origin, either deriving from the skin microbiota or external contamination. The decrease of UA below the regular biological range is caused by the ability of those bacterial species to metabolize UA to 5-hydroxyisourate through the action of the enzyme uricase, which is absent in humans.^43^ In such conditions, a multibacterial infection could lead to the formation of biofilms, boasting increased antibiotic resistance, making the wound prone to chronitization,^44^ and more susceptible to oxidative stress, as UA acts as a powerful reactive oxygen species scavenger.^45^ On the other hand, an increase in UA concentration above the normal range can be correlated to imminent or ongoing necrosis of the tissues, as a large number of cells die, releasing adenosine triphosphate (ATP) into the environment, which then degrades to UA.^46^ Continuous, real-time monitoring of UA in wound exudate can be a powerful diagnostic tool capable of providing useful medical intel in regards to the various wound-healing stages and thus the well-being of the patient. Few devices have been developed in the last years to address this issue: in the work performed by Kassal^47^ et al., a smart bandage based on a screen-printed three-electrode amperometric device was developed using Ag/AgCl and Prussian blue carbon inks for the fabrication of counter and working electrodes, the latter being modified with the enzyme uricase. The device was also integrated with a wireless unit, simplifying the recovery of the medical data; a similar approach^48,49^ was followed by Liu et al. and RoyChoudhury et al., who developed amperometric sensors for the enzymatic detection of UA through the embroidering of carbon-coated polyester yarns on medical gauzes and screen printing of a carbon-based paste on vinyl adhesive supported on textile wound dressings, respectively. All of these devices proved to be selective toward the detection of UA in the tested conditions, displaying a response range useful for the quantification of this analyte in wound exudate. However, the aforementioned devices involve the use of an enzyme to achieve sensitivity and selectivity, which contributes to an increased cost of manufacturing and more delicate handling conditions, along with a three-electrode cell design which entails the necessity to have a reference or pseudo-reference electrode, bearing potential cons (i.e., fragility, susceptibility to potential drifts, chemical resistance, biological fouling). A way to circumnavigate these issues is to adopt a different device architecture based on organic electrochemical transistors (OECT). This category of sensors relies on the interactions between a channel constituted by an intrinsically conductive polymer (ICP), such as poly(3,4-ethylenedioxythiophene):polystyrene sulfonate (PEDOT:PSS), and a gate electrode, which can be made by employing ICPs as well as other conductive substrates such as gold or platinum.^50^ The peculiarity of these materials lies in the presence of a conjugated π-system along the PEDOT backbone, which permits the formation of holes (namely polarons and bipolarons) that act as charge carriers, leading to very high conductivities, ranging up^51^ in the order of 10^3^ S/cm. Therefore, a variation in the concentration of holes in the polymer causes a sharp change in its electrical conductivity—this can be achieved by externally varying the gate potential or by redox reaction taking place at the gate electrode—allowing OECTs to be used as sensors, provided the existence of a suitable transduction mechanism for the analyte of interest. The previously mentioned phenomena are at the basis of the transistor behavior of OECTs, as the modulation of channel conductivity allows for an elevated signal amplification—since a small change in gate potential causes a great variation in drain current and strong signal noise filtering effects.^52^ Overall, these devices have been proven to be particularly versatile, as they can be fabricated on different substrates such as glass,^53^ poly(ethylene terephthalate),^54^ and textile threads^55^ for the detection and quantification of different analytes of biological relevance like glucose, lactic acid, uric acid, dopamine, ascorbic acid,^56,57^ or chloride ions.^58^ OECTs do not require the presence of a reference or pseudo-reference electrode, thus easing the manufacturing process and increasing the device robustness. Moreover, OECTs benefit from the aforementioned signal intrinsic amplification and filtering effect, dramatically improving the quality of the electrochemical data recorded and also requiring low voltages (<1 V) and absorbing low power (<1 W). Recent examples of OECTs developed for the detection of UA in wound exudate can be found in the work performed by Galliani^59^ et al., where a PEDOT:PSS-based OECT was fabricated on PET by means of screen printing. Signal transduction was again achieved by immobilizing the enzyme uricase in a dual ionic gelatin layer to reduce interference by negatively charged compounds. Selective amperometric response to variations in UA concentration in artificial wound exudate was reported in the biological range of interest, although the OECT was not designed to be wearable or to perform in-situ analysis. In this regard, a fiber-based OECT with wearable characteristics has been presented in the work of Tao^60^ et al. by employing cotton fibers coated with PEDOT and reduced graphene oxide (rGO). In this case, selectivity toward UA detection was achieved not by enzymatic transduction but rather upon gate functionalization with a molecularly imprinted polymer (MIP) membrane permeable to UA, even though the reported response range appears to be more limited when compared to the average upper-level UA concentration in wound exudate (1 nM–500 μM vs 220–750 μM). To address the various critical issues regarding the state-of-the art for the wound care system, we present in this work a novel, smart, textile wound dressing for the detection of UA in wound exudate based on an OECT configuration that employs a label-free transduction mechanism, offering device stability, repeatability of the high sensitivity and high selectivity toward the most common compounds found in wound fluid. The sensor is fully assembled using medical-grade textile materials and absorbing foams, constituting a passive and non-invasive sampling system that permits to carry out UA potentiostatic determination in flow conditions. Finally, in accordance with the Internet-of-Things and Point-of-Care analysis principles, an Arduino-based instrumental setup was developed to act as a supply unit for the integrated textile OECT sensors and allow the recorded data to be wirelessly transmitted to a smartphone using a custom-made application. Together with the simple but effective dressing architecture, this type of instrumental approach significantly impacts the overall device compactness, wearability, and low cost and offers a user-friendly interface. In fact, the smartphone app may lead to better and faster data collection, storage, and analysis among medical personnel, improving their ability to tailor therapies and treatments based on the patient’s needs.

## Experimental Section

### Chemicals and Buffers

Clevios PH1000 suspension (PEDOT:PSS) was purchased from Heraeus. (3-Glycidyloxypropyl)trimethoxysilane (GOPS), ethylene glycol (EG), potassium dihydrogen phosphate, potassium hydroxide, boric acid, sodium chloride, potassium chloride, potassium nitrate, urea, d-(+)-glucose, sodium l-lactate, albumin, alkaline phosphatase (ALP), and lactic acid were purchased from Merck. Uric acid (UA) was obtained from Fluka. Acetic acid and phosphoric acid were purchased from Carlo-Erba. Guanine, xanthine, and hypoxanthine were purchased from Sigma-Aldrich, Adenine was obtained from Alfa Aesar. Silicone elastomer and curing agent for the preparation of polydimethylsiloxane (PDMS) were obtained from Sylgard. The conductive silver paste was obtained from RS Components. All chemicals used were of reagent grade or higher. The phosphate buffer solution (PBS) was created from 0.1 M KH_2_PO_4_ while adjusting the pH to a value of 7.00 by adding 1.0 M KOH dropwise. The universal buffer (UB) was made by mixing 0.01 M H_3_PO_4_, 0.01 M H_3_BO_3_, and 0.01 M CH_3_COOH in KNO_3_ 0.1 M. The pH of the UB solution was corrected to pH values of 4.50, 9.00, and 10.5 by dropwise additions of 1.0 M KOH. Simulated wound exudate (SWE)^41^ was prepared by mixing 0.005 M KCl, 0.009 M urea, 0.002 M d-(+)-glucose, 0.009 M l-lactic acid, 22 g/L albumin, and 84 U/L ALP in a pH 7.00 solution of 0.1 M NaH_2_PO_4_ as a pH buffer and source of Na^+^ ions. The conductive textile thread is stainless steel based and was acquired from SparkFun Electronics (27 Ω/m, diameter 0.12 mm). A015THI and A030THI were obtained from Don & Low Ltd, Jettex 1005 was obtained from OR.MA. S.R.L., Royal 100 was obtained from Tenowo Italia S.R.L., LN 0084 was obtained from Kemex, PHT 3093 hydrophilic polyurethane foam was provided by the Freudenberg Group, and the heat-activated adhesive web glue was obtained from AB-Tec.

### Apparatus

Potential-controlled measurements were conducted using a CH Instruments 900B bipotentiostat for classical three-electrode cell setup, OECT-based potentiostatic determinations, and OECT-based flow injection analysis. Simultaneous potential-controlled and open-circuit potential (OCP) measurements were conducted by coupling a Keysight B2902A Precision Source/Measure Unit (for 2-channel *i*–*t* measurements) to a CH Instrument 660C potentiostat (for OCP-t measurements). Electrode potentials were applied against an aqueous saturated calomel reference electrode (SCE) while using a Pt wire as a counter-electrode (CE) when needed. All solution-based pH measurements were conducted using a combined glass electrode (Amel 411/CGG/12) connected to a pH meter (Amel instruments 338). Flow injection analyses were conducted using a LabFlow 1000 model high-performance liquid chromatography (HPLC) membrane pump connected to a 0.25 mm internal diameter capillary tube. Solid-state pH measurements were instead made using a flat gel membrane combined pH electrode (Hanna Instruments HI1413B). Textile OECTs hot-gluing was performed using a 2-STAMP 23 × 30 cm, 600 W temperature-controlled hot-press. All measurements were conducted at room temperature and pressure unless specified otherwise.

### Characterization of the Textile Materials

The various textile materials available were characterized to better understand their rheological-physical properties and thus to optimize their use in the fabrications of the textile sensors. Samples of the materials (approximately 4 cm^2^ sections) were weighed before and after being submerged for 2 h in distilled water. The water absorbing capacity (WAC) and time (WAT) were calculated according to the ISO 20158:2018 procedure using 4 cm^2^ square samples. The grammage of each material was also calculated by dry weighing 1 cm^2^ samples. The water vapor transmission rate (WVTR) was determined in compliance with the ISO 2528:2017 gravimetric procedure. Also, the pH of the materials was measured using a solid-state combined pH electrode. Ultimately, portions of the textile materials were saturated with distilled water and freeze-dried at 4 Torr for 2 days. The resulting samples were characterized by scanning electron microscopy (SEM, Cambridge Stereoscan 360) using an acceleration voltage of 20 kV and a current probe of 32 pA.

### Fabrication of the Textile Sensors

**Figure 1:**
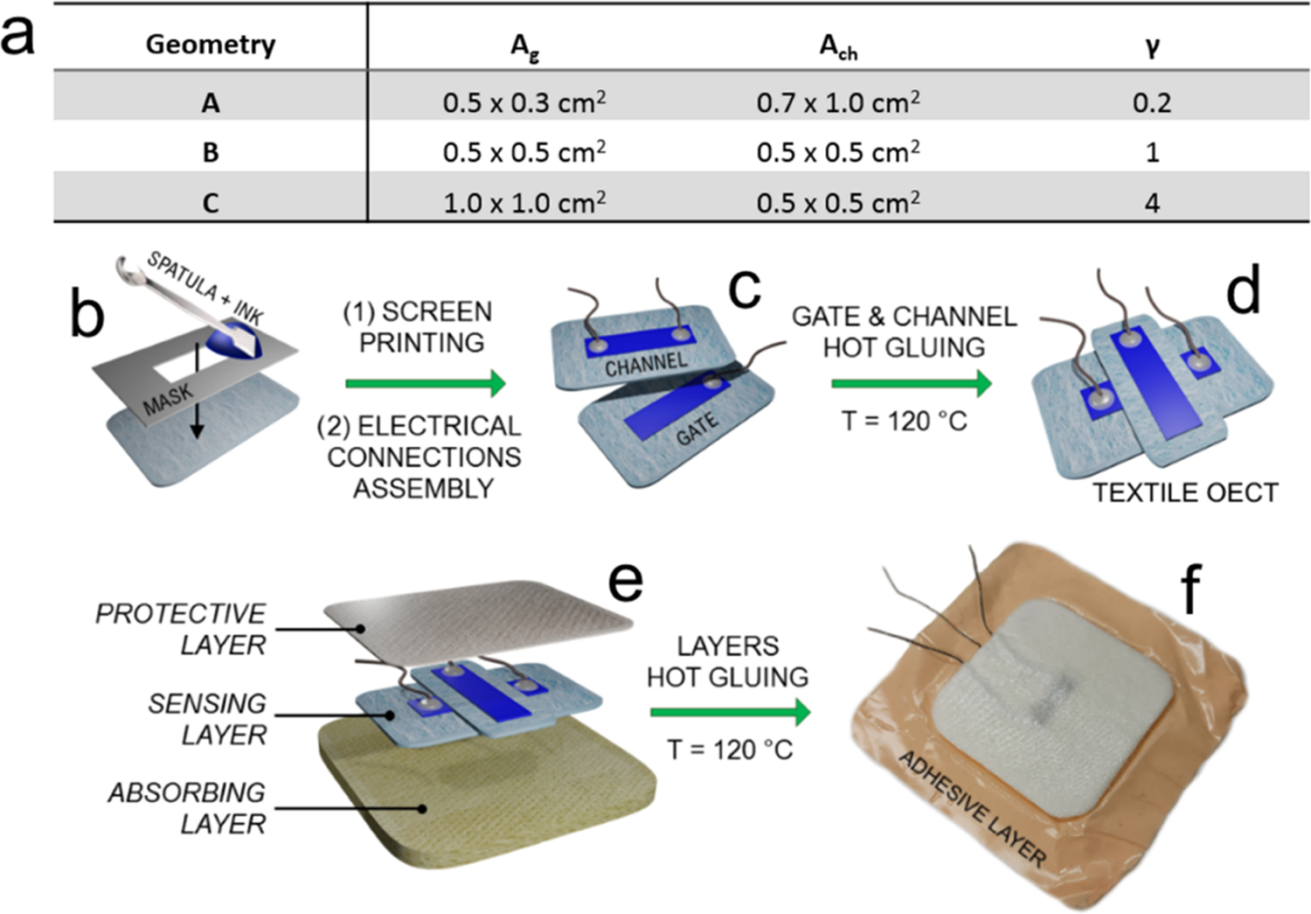
(a) OECT geometries featuring gate and channel electrochemical area absolute values and ratio. (b) Screen printing on a medical-grade textile substrate. (c) The aspect of the textile gate and channel upon assembly of the electrical connections and (d) hot-glued textile OECT. (e) Structure representing the components making up the smart OECT dressing. (f) The real aspect of a factory-assembled textile device.

Based on the optimized procedures developed and tested in our previous works,^25,26,61,62^ a conducting ink made of 78%_v/v_ PH1000, 20%_v/v_ EG, and 2%_v/v_ GOPS was mixed thoroughly and put inside an oven at 60 °C until 40% of the initial weight had been lost. The ink was then removed from the oven and cooled down to room temperature. Suitable masks were used to screen-print the desired sensor patterns on the sterile dressings (as described in Figure 1a). One milliliter of the conductive ink was applied to the mask, and three spatula streaks were performed to transfer the ink onto the medical gauze through the mask pattern. The functionalized textile materials were then dried in an oven at 60 °C, and electrical connections were created by sewing commercial conductive threads on the screen-printed PEDOT:PSS-based ink area. Afterward, a small amount of conductive silver paste was applied to minimize the contact resistance. The textile devices were then put on a hotplate at 150 °C for 10 min to allow partial reticulation of PEDOT:PSS chains by GOPS. Simultaneously, the textile electrical connections were insulated by applying a mixture of PDMS-curing agent (9:1 w/w). Once the PDMS cured properly, the devices were removed from the hotplate and cooled down to room temperature. Three different device geometries were tested to investigate the effect of the gate-to-channel area ratio on the sensors’ performance. This geometrical parameter is better represented as “γ”, and it is calculated according to eq 1
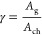
1Where *A*_g_ and *A*_ch_ are the gate and channel areas, respectively. The geometry data are summarized in Figure 1a. The smart textile sensors were then assembled (a scheme of the fabrication process is shown in Figure 1b–f) with an absorbing layer at the bottom and a protective layer at the top, using a special poly(vinyl alcohol)-based gauze by means of hot-pressing.

### Textile OECT *I*/*V* Characterization

To assess the correct behavior of the textile sensors as transistor, transfer and output curves, whose parameters were adapted from our previous works,^53,63^ were plotted in a 0.1 M PBS electrolyte (pH = 7.00), as represented in Figure 3b,c. Transfer curves were obtained by imposing a fixed drain–source (*V*_ds_) voltage bias equal to −0.3 V while linearly sweeping the gate–source voltage (*V*_gs_) from 0.0 to 1.0 V at a speed of 10 mV/s. Output curves were instead produced by sweeping *V*_ds_ from 0 to −0.7 V at a speed of 50 mV/s and stepping the *V*_gs_ from 0 to 0.8 V at 0.2 V intervals between each output curve. During all of the characterization, the drain (*i*_d_) and gate (*i*_g_) currents were also recorded.

### Potentiostatic Measurements in Flow Conditions

The integrated textile OECTs were subjected to flow injection analysis (FIA) using a LabFlow 1000 HPLC membrane pump to simulate the secretion of wound exudate. The textile OECT was connected to the bipotentiostat, applying a drain–source voltage equal to −0.3 V and a gate–source voltage of +0.6 V. The delivery capillary (possessing an internal diameter equal to 0.25 mm) of the HPLC pump was put perpendicular to the surface of the sensing layer of the textile OECT. First, uric acid solutions at known concentrations in PBS buffer were fed at a flow equal to 0.05 mL/min, then FIA tests were carried out in synthetic wound exudate. Before supplying a solution at a different UA concentration, the pump was purged with the next solution for 110 s at a flow equal to 1.6 mL/min.

### Implementation of Sensor-to-Smartphone Bluetooth Connectivity

An instrumental setup was developed based on an Arduino BLE programmable microcontroller, 16-bit ADCs and external adjustable dropout voltage regulators, designed to record and transmit via Bluetooth the current flowing in the OECT and to supply a *V*_gs_ = +0.6 V and a *V*_ds_ = +0.3 V. A custom-made smartphone application was developed to receive the collected data and plot in real time the sensor response to UA additions, which were performed by pipetting 2 mL dropwise onto the textile OECT as a proof-of-concept test.

### In Vitro Cytotoxicity Trials

In vitro cytotoxicity of the sensing layer consisting of the printed PEDOT-based ink on Royal 100 was evaluated by an accredited laboratory (Eurofins BioPharma Product Testing) following the ISO 10993-5:2009 method using mammal fibroblasts BALB/3T3 clone A31 (ATCC; CCL163) cell line. The experimental design included two 12-well plates containing a subconfluent cell monolayer. Supplemented culture medium was replaced with 1.2 mL of fresh supplemented culture medium, and the test sample and controls were added, while the blank wells were replaced with only 1.2 mL of fresh supplemented culture medium. The plates were incubated in an incubator at (37 ± 1)°C in (5 ± 1)% CO_2_ atmosphere for 24 h. For qualitative analysis, after 24 h, the plates were observed under an inverted microscope, and biological reactions were evaluated following a 0 (none) to 4 (severe reactivity) scale (according to ISO 10993-5:2009). For quantitative analysis (optical density), after microscopic observation, each well was emptied, washed with Dulbecco’s phosphate buffer solution, and treated with neutral red medium for 3 h at (37 ± 1)°C in (5 ± 1)% CO_2_ atmosphere. Subsequently, the neutral red medium was removed, and each well was rinsed with Dulbecco’s phosphate-buffered saline (DPBS). The plates were dried, the desorb solution was added, and the plates were incubated for at least 15 min at room temperature with gentle agitation to form a homogeneous solution. Optical density was measured at 540 nm by Gen5 software (Biotek) using a microtiter plate reader. For the interpretation of the results, the achievement of a numerical grade greater than 2 and a cellular viability reduction of more than 30% is considered a cytotoxic effect.

## Results and Discussion

### Textile Material Characterization

**Figure 2:**
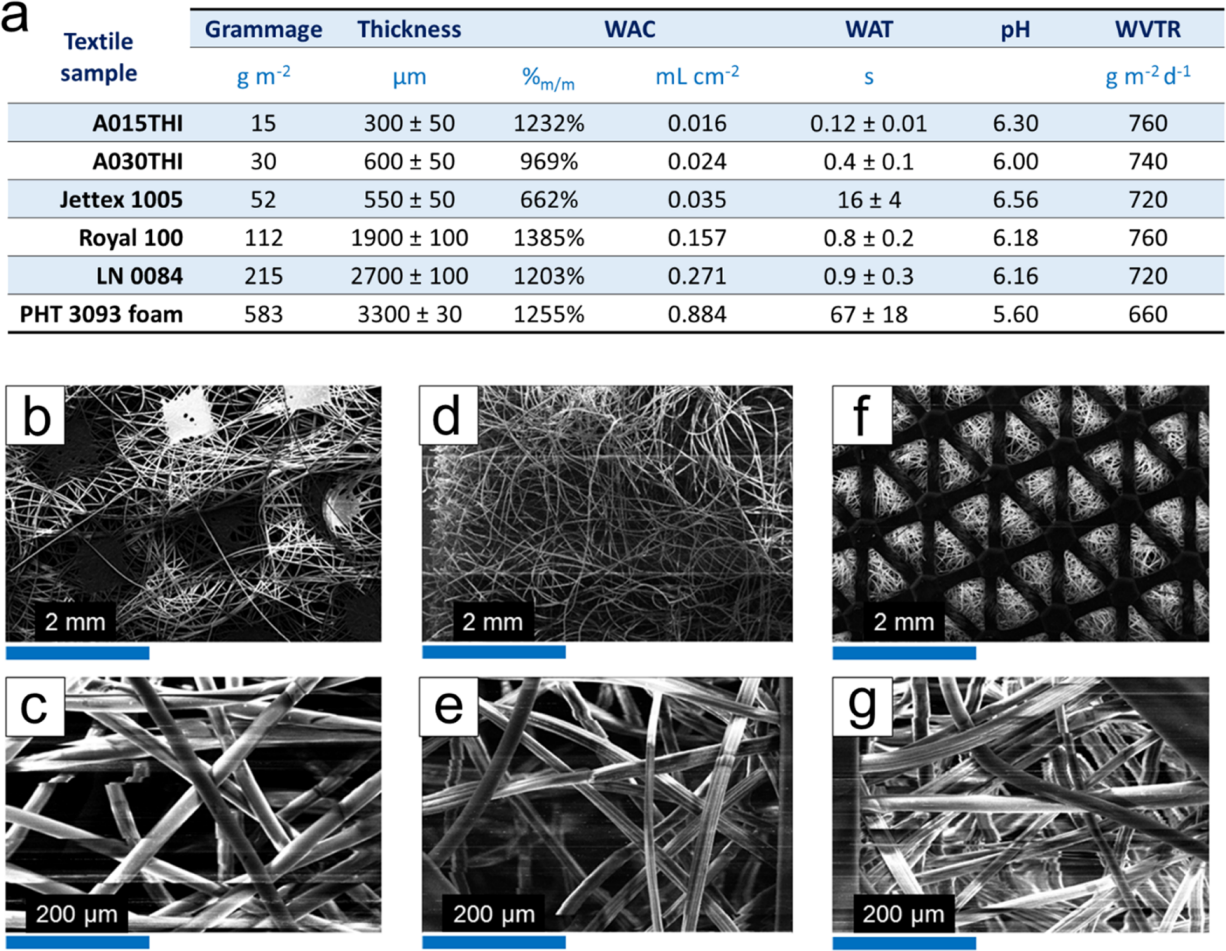
(a) Textile material characterization results, the WAC value is provided by mass and by surface. SEM imaging of the textile materials: (b, c) A015THI, (d, e) Royal 100, and (f, g) LN 0084.

Since the development of smart dressings can exploit a broad plethora of high-tech medical-grade materials, profound knowledge of their functionalities plays a key role in the effective design of performing devices. A set of nonwoven, medical-grade dressings exhibiting different morphological and physicochemical properties were thoroughly investigated in this work. The resulting parameters are reported in Figure 2a. A015THI (Figure 2b,c) and A030THI (Figure S1a,b) are both based on electrospun hydrophilic polypropylene, Jettex 1005 (Figure S1c,d) is made from polyester fibers, while Royal 100 (Figure 2d,e) and LN 0084 (Figure 2f,g) are both composed of polyester and Rayon. PHT 3093 hydrophilic polyurethane foam (Figure S1e,f) is instead made from polyester fibers coupled with polyurethane foam. All materials were first characterized in terms of grammage, water absorbing capacity (WAC), water vapor transmission rate (WVTR), water absorption time (WAT), surface pH, and morphology. The sample grammage is quite variable, as it is correlated with the type and thickness of the textile material, as well as the packing density of the nonwoven fibers. WAC is a relevant parameter to evaluate the water retention and swelling capacity of textile materials, which are major criteria in choosing the proper dressing depending on the wound exudate level and exuding rate.^64^ The WAC by mass values are similar among the different samples, as they are mainly associated with the absolute weight that is intrinsically normalized during the calculation of this parameter. In this case, Jettex 1005 exhibits the lowest WAC by mass, as it presents more tightly packed and less hydrophilic fibers, thus leading to lower porosity and allowing for lower retention of water. Instead, adopting the WAC by surface, a clearer picture emerges for the different textile materials, as the reported values are directly correlated with the grammage. Moreover, it is known that a smaller fiber diameter originates a higher surface-to-volume ratio that improves the water retention capacity.^65^ Here, the diameter of the fibers was calculated from SEM images and can be correlated with the WAC by surface. A015THI and A030THI, with the largest fiber diameter among the materials under investigation (20 ± 1 μm) and the smallest grammage, present the lowest WAC by surface values. With a higher grammage and a slightly smaller fiber diameter of 16 ± 3 and 17 ± 6 μm, Royal 100 and LN 0084 show improved WAC values of 0.157 and 0.271 mL cm^–2^, respectively. Differently, the densely packed fibers in Jettex 1005, despite having the smallest diameter (11.3 ± 0.7 μm), again strongly limit the WAC by surface to a value of 0.035 mL cm^–2^ that is slightly higher than A015THI and A030THI. Finally, PHT 3093 Foam outperforms all of the materials under investigation with the highest water retention capacity, thanks to the combination of a fibrous layer and a foam having pore diameters ranging from 190 ± 30 to 350 ± 100 μm. The WAT values obtained during these tests are influenced by both the type of material and its morphology. A015THI and A030THI report the lowest WAT values, as they are the materials with the lowest grammage. Analogously, Royal 100 and LN 0084 display similar values since they are constituted of the same type of fiber, the latter having a slightly higher value due to the increased thickness. Interestingly, Jettex 1005 shows a WAT value higher than expected. Despite being thinner than Royal 100, its fibers are more tightly packed, preventing efficient water absorption. Finally, PHT 3093 Foam reports the highest WAT, being a non-fibrous, bulky material. The surface pH measurement of textile samples was performed by wetting the material with a few drops of deionized water and subsequently applying the solid-state combined pH electrode. All of them presented comparable values with an average pH of 6.24, with the exception of PHT 3093 Foam (pH 5.60). This can be attributed to the different chemical nature of the fibers constituting the material, as polyurethane-based materials could present terminal carboxylic acid groups. The last parameter under investigation was the WVTR. Due to the random arrangement of the fibers, pores of all geometrical shapes can be present in nonwoven textiles where a higher porosity promotes permeability, which in turn decreases with sample’s thickness and density.^66^ WVTR plays an essential role in moisture control at the wound bed that is complementary to WAC. In fact, a high WVTR determines a superior degree of permeability and breathability; however, optimal WVTR values should be chosen according to the wound type to avoid both dehydration (too high WVTR) and maceration (WVTR lower than normal skin,^65,67^ e.g., 204 g m^–2^ d^–1^). By comparing the WVTR values, it is evident that all of the fibrous materials exhibit similar WVTR, with an average value of 740 g m^–2^ d^–1^. In contrast, PHT 3093 Foam, which is the only material with an additional non-fiber-based component that hinders the passage of water vapor and lowers the breathability of the material, shows the lowest WVTR. Additional WVTR measurements were performed on the textile materials chosen for the OECT fabrications during the various production steps. Upon conductive ink screen printing, a Royal 100 channel displayed an increase in WVTR equal to 2% when compared to the pristine gauze. Instead, the assembled OECT (featuring the hot-glued gate and channel) exhibited a 12% decrease in WVTR. The same value was observed for the fully assembled device, incorporating the absorbing layer composed of LN 0084.

### Design and Characterization of the Textile OECT

**Figure 3:**
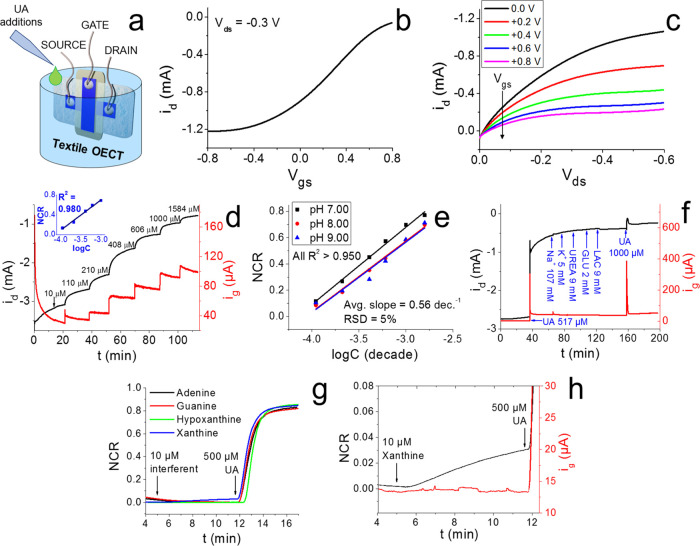
(a) Instrumental setup and textile sensor cross-like geometry (geometry B, γ = 1) regarding the analysis conducted in this figure. (b) Transfer and (c) output curves obtained in a 0.1 M PBS electrolyte (pH = 7.00). (d) Instrumental response for a potentiostatic *i*–*t* determination of UA in 0.1 M PBS at pH 7 and its resulting calibration curve. (e) Investigation of the pH influence on the device sensitivity. (f) Selectivity test. (g) Selectivity test comprising the most common purines at their maximum biological concentration in wound exudate. (h) Zoomed xanthine addition. All *V*_ds_ = −0.3 V; all *V*_gs_ = +0.6 V. All error bars are smaller than the data points.

Once the textile materials were fully characterized, the attention was first focused on the sensing layer. This layer is intended to host the OECT-based sensor for UA and should (i) guarantee the desired printing resolution of the OECT elements, (ii) mechanically support the sewing of the textile electrical connections and foresee future assembly toward in flow monitoring, and (iii) exhibit intermediate WAC values to allow the fluid to freely pass across the sensing layer without giving stagnation or backmixing. For these reasons, Royal 100 was identified as the best option exhibiting intermediate grammage and WAC by surface values, and especially providing a higher screen-printing resolution (as depicted in Figure S2) and easier sewing with respect to Jettex 1005, which was the other selected material for the construction of the sensing layer according to its WAC and WVTR values. Therefore, the textile OECT components (i.e., gate and channel, referring to the step depicted in Figure 1c) were screen printed on Royal 100 following the procedure described in the Experimental Section. Afterward, the screen-printed OECTs were electrochemically characterized while submerged in 0.1 M PBS at pH 7, as represented in Figure 3a. The sigmoid *i*_d_ vs *V*_gs_ shape obtained in the transfer curves in Figure 3b can be explained through the electrochemical processes taking place in the OECT system, caused by the variation of the gate voltage, according to the doping–dedoping reactions described here
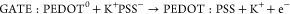
2
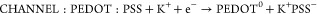
3An increment of *V*_gs_ causes a positive polarization of the gate electrode, which induces the oxidation of the neutral PEDOT units. The electrons removed (constituting the gate current *i*_g_) are then injected into the channel, causing the reduction of charge carriers delocalized in the PEDOT:PSS. This process is accompanied by the insertion of potassium ions from the electrolyte into the channel by the repulsive action of the gate electrode. Therefore, a *V*_gs_ increase causes a decrease in the channel’s electrical conductivity, as it is proportional to the concentration of electron holes. This change is transduced in a decrease of *i*_d_, as reported in Figure 3b. The reverse process takes place in the case of negative *V*_gs_. The same doping–dedoping principles are valid when producing output curves (Figure 3c), where the stepped increase of *V*_gs_ provokes an overall decrease in *i*_d_ among the recorded curves, as the same electrochemical phenomena described in Reactions 2 and 3 are taking place. Therefore, according to the experimental evidence, it is safe to state that the textile OECT described here presents a behavior analogous to the more classic thin-film PEDOT:PSS-based organic electrochemical transistors^53^ displaying a clear signal (current) modulation upon the variation of the gate voltage. More importantly, this comparison can also be made with the transfer curves of a fully textile OECT from the work of Gualandi^61^ et al. In both cases, for transfer curves, device turn-off occurs between *V*_gs_ 0.8 and 1.0 V, regardless of the absolute *i*_d_ value, which is mainly correlated with the electrical resistance of the device, each one being different due to the different techniques and materials involved in their construction. In addition to a comparison with similar devices, the OECT geometry was also taken into account while performing *I*/*V* characterization. By analyzing the output and transfer curves in Figure S3 for each geometry tested, it can be seen that an increasing gate electrode area allows for a stronger signal modulation of the drain current for the same applied *V*_gs_, as seen in the output and transfer curves. However, considering the desired future integration of the textile OECT printed in the Royal 100 sensing layer within a fully assembled smart dressing operating in flow conditions, a planar transistor architecture is not convenient. In fact, the textile OECT should operate efficiently also in the case where an exudate flow in the order of μL/min acts as the electrolyte solution. For this reason, the textile OECT architecture was modified to display for the first time a cross-like geometry by joining the gate and channel components with a layer of permeable web glue through hot-pressing (as described in Figure 1d). This process serves multiple purposes: it first creates a physical bond between the OECT parts, leading to a more sturdy and robust sensor; second, it greatly reduces the volume needed to be retained in the OECT to maintain a constant gate-to-channel electrolytic contact, which is particularly useful when operating in flow conditions in which less solution is needed during the device testing. Finally, the permeable web glue layer represents an additional insulating layer preventing any short-circuit between the gate and channel. Being a new type of OECT configuration, the cross-like geometry devices were tested under the same conditions relative to Figure S3 to perform *I*/*V* characterization (reported in Figure 3), where the same type of response was obtained when compared to the unassembled OECT, although with a stronger signal modulation, which can be attributed to a smaller electrolyte electrical resistance, as the gate-channel distance is minimized in the cross-like geometry.

### Electrochemical Response of the Textile OECT to Uric Acid and Interfering Species

**Figure 4:**
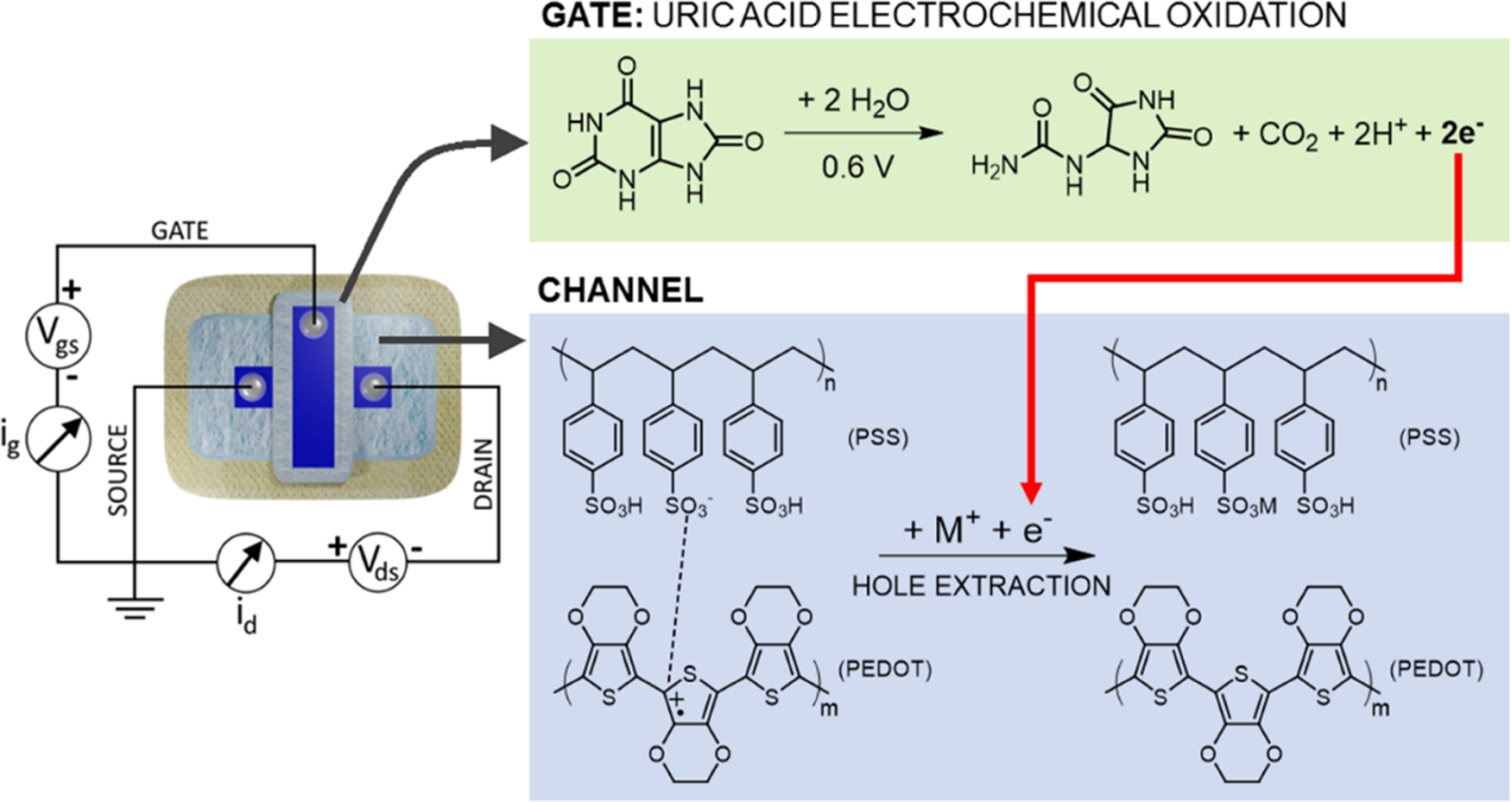
Scheme depicting the OECT circuital setup employed for the potentiostatic UA determination and the relative chemical and electrochemical reactions taking place at the gate electrode and channel.

The cross-like textile OECTs were preliminarily tested in an electrochemical cell to assess their response to uric acid, whose involved electrochemical reactions are reported in Figure 4. The analyses were performed by submerging the device in 0.1 M PBS at pH 7.00 under magnetic stirring while performing progressive additions of UA (setup in Figure 3a). The potentiostatic determination was carried out upon application of constant voltages, i.e., *V*_gs_ = +0.6 V and *V*_ds_ = −0.3 V, chosen on the basis of a correlation study between gate voltage and NCR sensitivity on glass-based OECTs, which is reported in Figure S4. The corresponding *i*_g_ and *i*_d_ flowing in the system were then measured over time. Under the reported electrochemical conditions, the *V*_gs_ is sufficiently positive to induce the irreversible electro-oxidation of UA, as proved by measurements of OECT electrochemical potential (*E*) using a saturated calomel electrode connected to the source terminal. It is possible to notice (Figure S5a) that an applied *V*_gs_ of +0.6 V brings the *E*_g_ to a value equal to +0.7 V vs SCE, which is well above the oxidation peak potential obtained for uric acid at any pH tested (Figure S5b,c). The electrochemical oxidation of UA generates its derivative allantoin, carbon dioxide, hydrogen ions, and two electrons.^68^ These electrons are then injected into the channel through the external circuit, along with a cation available in the electrolyte, causing PEDOT:PSS reduction and the change in the channel’s electrical conductivity. The preliminary instrumental response arising from a potentiostatic determination of UA in an electrochemical cell for a Geometry “B” OECT (γ = 1) is reported in Figure 3d, where a net *i*_d_ decrease is clearly visible for every progressive addition of UA. At the same time, a proportionality between the *i*_g_ and UA concentration is observed, as the gate current is related to the number of UA molecules oxidized per time unit, which in potentiostatic conditions correlates with analyte concentration. The calibration plot for the potentiostatic determination of UA is represented as an inset in Figure 3d. The drain current was chosen as the designated analytical parameter since, in OECT systems, it is the signal with the highest signal-to-noise ratio and the highest absolute value, making its recording instrumentally reliable and analytically relevant. The *i*_d_ values were converted into normalized current response (NCR), calculated according to eq 4
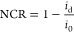
4where “*i*_d_” is the drain current recorded for a generic steady state and “*i*_0_” is the drain current relative to the steady state reached at a UA concentration equal to 10 μM (unless specified otherwise). This operation is performed to better compare the performance of different devices, as it mitigates the influence of the starting conditions of the electrochemical system, such as the initial redox state of the PEDOT:PSS chains and the channel resistance. A clear linear dependence between the NCR and the base-10 logarithm of UA concentration is shown in Figure 3d, from 110 to 1000 μM and a mean response time of about 7 min (calculated as *t*_90_, i.e., the time for the signal to reach 90% of the intensity between two UA additions). This interval is more than sufficient for the purpose of detection and quantification of the concentration of UA in wound exudate, as the mean range reported in the literature is 220 ÷ 750 μM.^41^ Concurrently with UA gradients, pH shifts are known to occur along with the wound-healing progression.^69,70^ For this reason, additional potentiostatic determinations of UA in an electrochemical cell were conducted to investigate the influence of pH on the OECT sensitivity, as UA oxidation potential significantly shifts toward lower values as the pH increases (Figure S5c and d). Three determinations (Figure 3e) were thus performed consecutively on the same textile OECT at pH 7.00, pH 8.00, and pH 9.00, as these are the more commonly encountered values in the wound bed and wound exudate across the various healing phases. No significant sensitivity changes among the calibration plots were observed, as a relative standard deviation (RSD) of 5% was calculated. Moreover, no statistical differences exist within both the slope and intercept populations (*t*-test with *p* = 0.95), thus demonstrating that UA detection is not affected by pH variations in the optimized experimental conditions. To better investigate the device’s selectivity, a potentiostatic UA determination was conducted by performing single and separate additions of UA and some of the major components of human wound exudate at their typical concentrations.^41^ The OECT response is depicted in Figure 3f: a selective response to UA is clearly visible, as none of the interferents caused a significant change in the *i*_d_ ascribable to ionic strength variations or the occurrence of redox reactions. Following cell rupture, local accumulation of metabolites originating from the released adenosine triphosphate occurs in tissues and biofluids. These metabolites are mainly purines, such as xanthine and hypoxanthine, whose catabolism ends up with uric acid production, together with the release of reactive oxygen species.^46^ To date, no comprehensive study concerning the role of these metabolites in chronic wounds has been carried out, and the available literature reports propose contrasting interpretations about UA precursor accumulation at the wound bed.^46,71^ However, as these precursors are electroactive and structurally analogous to UA, a selectivity study was carried out to investigate the possible interference on the OECT sensor response originating from high adenine, guanine, xanthine, and hypoxanthine concentrations found in the exudate secreted by hard-to-heal wounds.^71^ The results are reported in Figure 3g and h. The oxidation waves ascribed to these purines have been resolved by differential pulse voltammetry at PEDOT-based sensors modified with nanomaterials,^72,73^ being guanine and xanthine the most easily oxidizable. In the selected experimental conditions (PBS, pH 7.00), only xanthine gives a detectable interference causing a 3% variation of the signal recorded at the OECT (zoom in Figure 3h). This may be explained considering its favorable interaction with the positively biased gate electrode, as xanthine, like UA, carries a negative charge at pH 7.00.^74,75^ Nevertheless, such a high xanthine level would lead to an error that is less than 5% in the estimation of UA concentration, thus highlighting the excellent performance of the textile OECT despite the absence of any specific biorecognition element or modifier to functionalize the sensor structure.

### Response of the Fully Assembled Smart Dressing to Uric Acid in Flow Conditions

**Figure 5:**
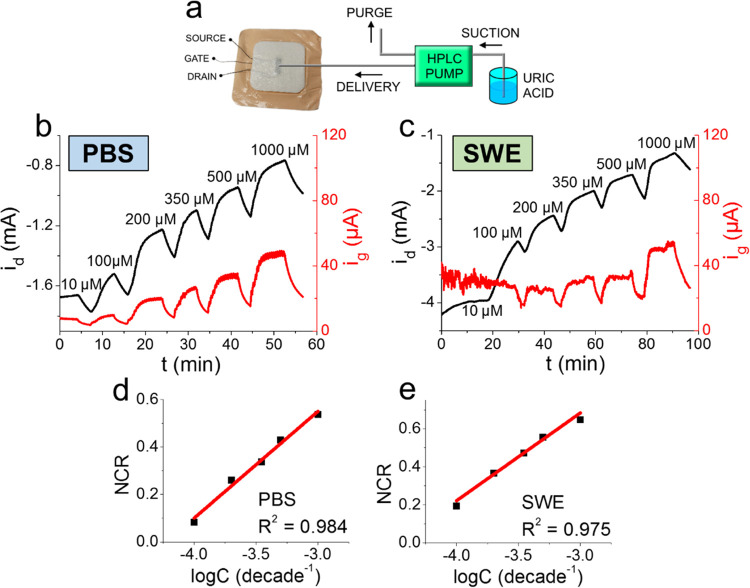
(a) Experimental HPLC setup used in flow analysis. (b) *i*–*t* response of a textile OECT in flow conditions to UA solutions in PBS. (c) *i*–*t* response of a textile OECT in flow conditions to UA solutions in synthetic wound exudate. (d) The calibration curve obtained in PBS–Slope = 0.45 ± 0.03 decade^–1^. (e) The calibration curve obtained in SWE–Slope = 0.47 ± 0.03 decade^–1^. All error bars are smaller than data points.

Once the transduction mechanism in the textile OECT is assessed through the previous characterization, which also preliminarily highlighted the intrinsic repeatability of the analysis and stability of the device, OECT sensors printed on Royal 100 were integrated with the absorbing and protective layers to perform potentiostatic determination of UA in flow conditions (setup in Figure 5a). Based on the results obtained during the characterization of the different dressings, the best materials for the protective and absorbing layers were identified as follows. A015THI was chosen for the protective layer, whose main function is to separate the wound from the sensing layer since its low grammage and low WAC promote the free flow of SWE toward the textile OECT sensor. As for the absorbing layer, it should act as a fluid reservoir and passively pump the exudate away from the underlying layers to avoid stagnation and allow for continuous wound fluid monitoring. Although PHT 3093 Foam exhibited the highest water absorption capability, it was also subjected to a significant mechanical deformation upon contact with the fluid that was detrimental to electrical connection stability. For this reason, LN 0084 was chosen as the absorbing layer. After full assembly, the smart dressing was tested: potentiostatic detection of UA in flow conditions was performed, imposing *V*_ds_ = −0.3 V and *V*_gs_ = +0.6 V. Data collected during the analysis display a clear response of the smart dressing upon increasing UA concentrations both in PBS and simulated wound exudate delivered at a flow of 0.05 mL min^–1^. The pump flow was set to this value with the aim of mimicking the exuding rate of a real hard-to-heal wound based on the original classification of Mulder^76^ and the more recent literature.^77^ As expected, *i*_d_ decreases as the UA concentration is increased. Among each subsequent UA concentration tested, a sharp decrease in both *i*_d_ and *i*_g_ was observed. This can be attributed to an almost quantitative oxidation of UA by the gate electrode when the flow of solution is stopped due to the synergistic effect of a large electroactive area and a small retained volume of solution in the sensing layer. The sensitivities of the device operating in PBS (Figure 5b) and SWE (Figure 5c), respectively, 0.45 ± 0.03 and 0.47 ± 0.05 decade^–1^, were evaluated by performing a two-tailed *t*-test (*p* = 0.95) to compare the slopes of the *i*_d_ calibration plots, proving that no statistically significant differences exist between the two sensitivities. Moreover, both sensors reported a linear range of response between 100 and 1000 μM UA, which is similar to the range obtained in the standard electrochemical cell and useful for the purpose of quantifying UA in wound exudate. Another feature of the current vs time graphs deserving attention is the difference between the *S*/*N* ratio achievable for *i*_g_ and *i*_d_ plots. Considering the response obtained in SWE relative to Figure 5c, the *S*/*N* ratio was found to increase from 51 to 122 from *i*_g_ to *i*_d_ steady-state currents reached at 100 μM UA. The value was calculated as the ratio between the average steady-state current (*n* = 100) and its standard deviation. An additional parameter on the performances of OECT-based sensors regards the signal amplification phenomena taking place among the gate and drain currents, better known as gain. It is calculated experimentally as the ratio between the variation of *i*_d_ and *i*_g_ (according to eq 5) for each steady-state current at every UA concentration tested. The final gain relative to a single sensor is presented as the average value.
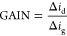
5A higher gain is thus associated with higher signal amplification and stronger filtering of the instrumental noise, which are two important parameters concurring in the quantification of the OECT sensing performances. This parameter will be further discussed in the following paragraph, where the geometric optimization of the device is reported. It is worth noting that baseline stability is successfully achieved also in flow conditions, as demonstrated by the low RSD% (equal to 1.2% h^–1^) calculated on the *i*_d_ recorded over a time of about 2 h. Following the delivery of each new UA concentration on the smart dressings in Figure 5, a signal plateau is reached in a few minutes in the currents recorded at the gate electrode, where direct UA oxidation takes place. Moreover, as soon as the flow of a solution containing the analyte is suspended to allow purging, the *i*_g_ baseline is quickly restored. Due to the interplay of electronic and ionic circuits that originates the amplified *i*_d_ response, the stabilization of the current recorded at the drain electrode (zoom in Figure S6) is slower but still guarantees a *t*_90_ of about 5 min only, and its correlation with UA concentration is independent of baseline restoration among subsequent UA administrations. All in all, these observations suggest the lack of memory effect and/or retention of the analyte at the sensing interfaces,^78^ which might occur due to a lowered permeability of the fully assembled dressing, thus confirming that the device developed here allows the reliable analysis of artificial exudate flux in real time.

### Textile OECT Architecture Optimization

As already mentioned, three different device geometries were tested to explore the influence of the gate and channel electrochemical active area and their ratio, γ, on the sensor performance. Table 1 reports the main analytical parameters of the three tested geometries when operating in static or flow conditions. Considering the results obtained in PBS, the OECTs possessing γ = 1 proved to be the most sensitive, reporting the highest slope alongside the lowest average response time. Instead, the highest average gain was obtained for γ = 0.2 OECTs, as a smaller gate electrode leads to a smaller gate current, resulting in an increased gain. Based on this information and also taking into account the scarce printing reproducibility obtained for the devices with γ = 0.2, the OECTs with γ = 1 were chosen to be tested in flow conditions in SWE. The NCR slopes reported in SWE were comparable with those obtained in PBS, as confirmed by the *t*-test (*p* = 0.95), proving that no significant statistical differences exist between the two conditions. The average response time was found to be higher in SWE than in PBS, presumably due to the increased viscosity of the solution caused by the high albumin content, which slows down the UA diffusion processes to the gate electrode. Consequently, a hindered diffusion causes less matter to be transferred to the electrode surface per time unit and, therefore, lower currents. The decrease in gate current also leads to an increase in the average gain, which was indeed found to be higher when confronted with that obtained in flow conditions in PBS. Conversely, the devices with the largest gate area (γ = 4) exhibit analogous sensitivity and gain if compared to the OECTs with γ = 1 but longer response time. Based on these comparisons and taking into account (i) the scarce printing reproducibility obtained for the devices with γ = 0.2, (ii) the larger fingerprint of the devices with γ = 4, bringing no significant advantages from the analytical performances point of view, and (iii) the shorter response time combined with high sensitivity possessed by the γ = 1 geometry, the latter was chosen as the best trade-off.

**Table 1:** Textile OECT Performances Reported in Electrochemical Cell (PBS) and Flow Conditions in PBS and SWE

	electrochemical cell (PBS)			flow (PBS)			flow (SWE)
γ (*A*_g_/*A*_ch_)	0.2	1	4	0.2	1	4	1
NCR slope (decade^–1^)	0.55	0.62	0.44	0.36	0.47	0.47	0.46
NCR slope error	0.06	0.03	0.01	0.02	0.03	0.03	0.03
*R*^2^ (NCR slope)	0.980	0.994	0.999	0.981	0.984	0.980	0.975
*t*_90_ (s)	476	439	575	1094	272	561	702
average gain	43	23	19	106	33	43	71

### Repeatability, Reproducibility, and Resilience to Mechanical Deformations

**Figure 6:**
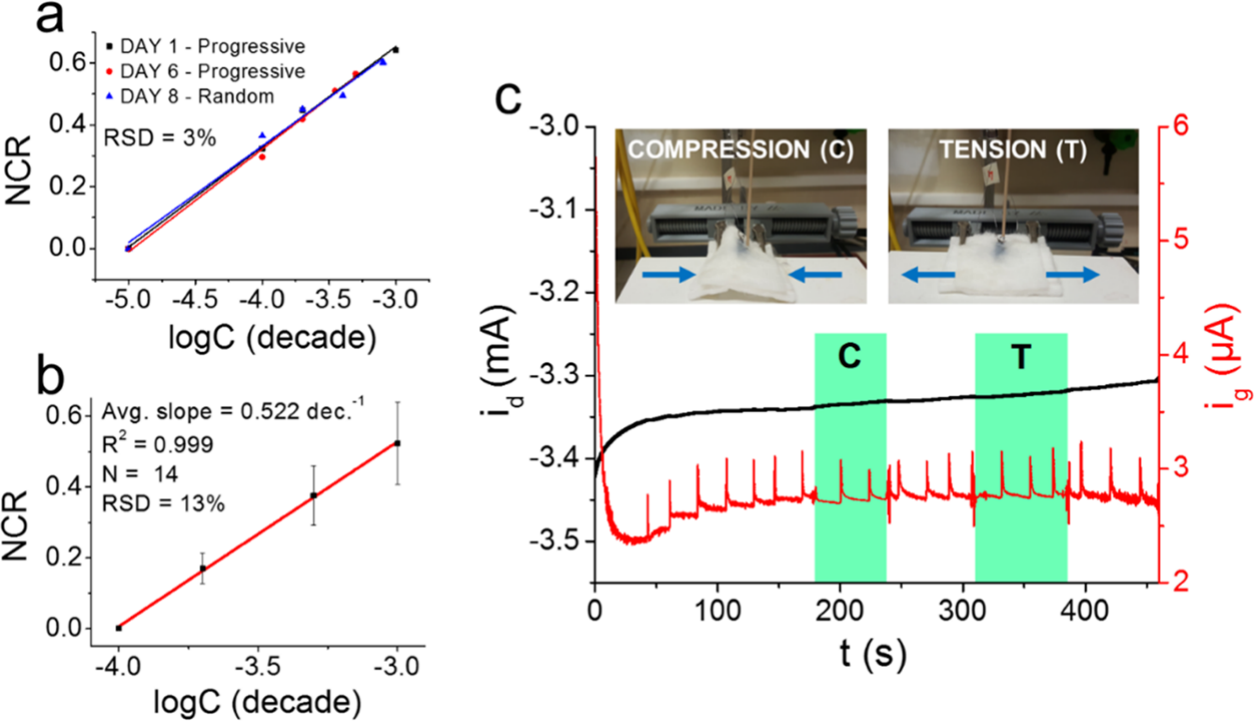
(a) Repeatability and (b) reproducibility trial results performed in 0.1 M PBS at pH 7.00 for a textile OECT (*V*_ds_ = −0.3 V; *V*_gs_ = +0.6). (c) The effect of mechanical deformations on the fully assembled dressing.

The results obtained in static and flow conditions demonstrated the intrinsic selectivity of the smart dressing toward UA, especially considering the flow analysis performed in SWE, for which the two-tailed *t*-test confirmed that no matrix effects are present compared to the flow analysis in PBS. To further estimate the device robustness and the reliability of the procedures involved in the textile OECT production, the repeatability and reproducibility of the sensor performances were also evaluated. The former was assessed by recording the signal variation caused at the same dressing upon FIA delivery of 200 μM UA during successive days (Figure S8) and by comparing three independent, full UA calibrations acquired in flow conditions in PBS using the same smart dressing (geometry B, γ = 1) over a period of 8 days, where the sensor was washed thoroughly and dried in the oven at 72 °C between each analysis. An RSD equal to 3% (Figure 6a) was obtained, and an important aspect regarding this experiment is the fact that a coherent response was achieved for the analysis performed by random additions on Day 8, which underlines the signal reversibility of the textile OECT in flow conditions (Figure S7). Furthermore, the extended lifetime of the devices (up to 8 days) is importantly well beyond the actual need for a real application since wound dressings are meant to be disposable and worn for limited periods of time. From the above-reported test, the utility of employing *i*_d_ as the analytical signal is also clear since *i*_g_ is highly affected by the noise, making it poorly suitable as sensing parameters. Finally, the reproducibility assessment (Figure 6b) was done by comparing the data obtained from 14 different OECTs tested in the same environment. An average slope of 0.52 decade^–1^ was calculated (in such a case, the *i*_d_ values were normalized at 100 μM UA as some sensors did not reach a clear steady-state signal at 10 μM concentration), with an associated RSD equal to 13%. The excellent repeatability and reproducibility obtained, together with the rather regular peak width and shape of the transient signals recorded before the steady state is reached, also suggest that the fully assembled dressing guarantees a reproducible and continuous fluid flow across the various layers without affecting mass transport or fluid rate.^79^ In light of these results, the solidity of screen printing as the chosen technique for device production also emerges, as it offers unique advantages with respect to other methods employed for PEDOT-based textile electrode fabrication, such as ink-jet printing or dip coating, including the fact that it is an already well-known and widely used printing technique in the fashion industry that can be easily automatized. On the one hand, ink-jet printing offers higher printing resolutions but introduces huge limitations in terms of fabrication throughput. On the other hand, dip coating improves the throughput, but the complete impregnation of all of the fibers occurs with a complete lack of control over the amount of deposited polymer.^80^ Conversely, the amount of polymer deposited on the fabric surface can be finely controlled using screen printing, and high reproducibility can usually be achieved upon optimization of the ink formulation and viscosity, as it will greatly affect the ink drop size and their interaction with the textile material, thus eventually impacting in the geometrical resolution and the shape reproducibility.

**Figure 7:**
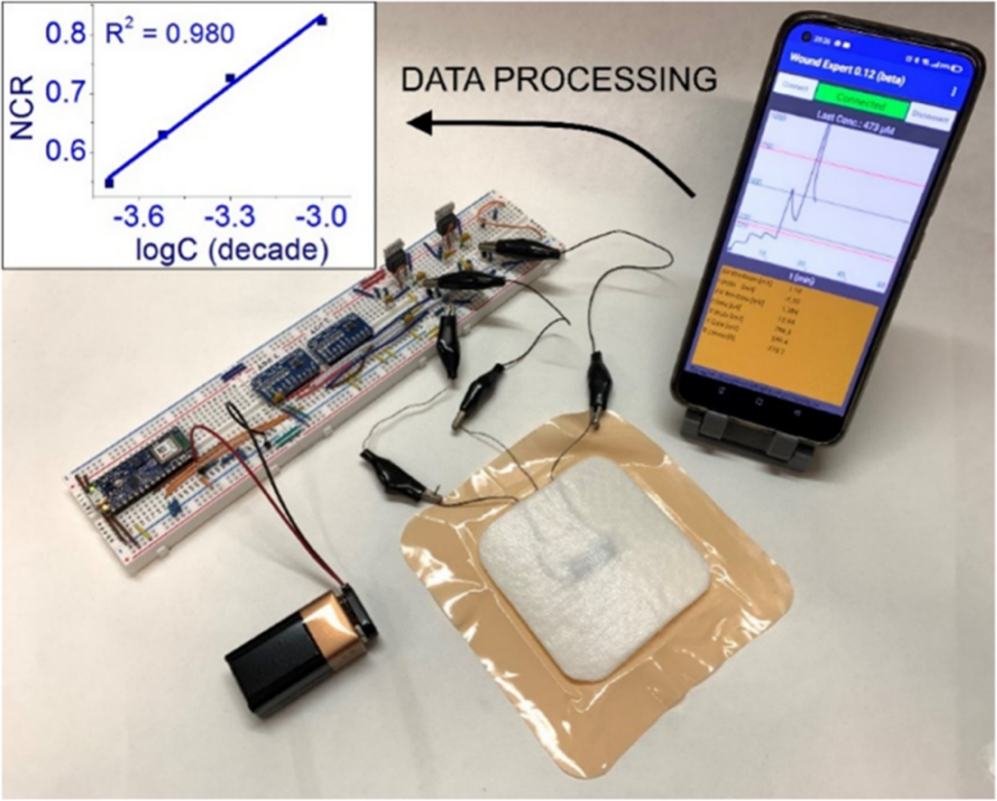
Experimental setup for the Arduino BLE potentiostatic determination, showcasing the circuitry, smartphone app, textile OECT, and its relative calibration plot (*V*_gs_ = +0.6 V; *V*_ds_ = +0.3 V).

An additional consideration concerning device’s reliability in the field of flexible electronics in the last decades regards their resiliency against deformation or different physical–chemical stimuli, as well as the stability of the electrochemical signal over time. These are currently major technological issues that should be addressed to keep the cost of the device low.^81^ In recent years, multimodal sensors have entered the IoT scenario exploiting the relationship among the materials’ properties and sensing mechanisms to decouple different stimuli and provide more accurate measurements.^82^ In particular, different deformations, such as stretching or bending, are likely to affect the electrical outputs of flexible electronic devices. To suppress such cross-sensitivity, several strategies have been reported, including the design of strain-insensitive geometries and patterns,^83^ integration of multiple sensing units with different sensing mechanisms,^84^ control of the active material concentration in the composite,^85^ and addition of fillers for mechanical reinforcement.^86^ For these reasons, we investigated the dressing response to compressive and tensile mechanical stimuli during FIA recordings at a rate of 0.05 mL/min. The fully assembled textile OECT was fitted on a custom-made 3D printed vise, and the gate/drain currents were measured upon application of the relative voltages (*V*_gs_ = +0.6 V, *V*_ds_ = −0.3 V). An image of the setup is reported in Figure S9, while the results are depicted in Figure 7c. Once the drain current reached a steady state, compressive and tensile forces were exerted on the device. Under compression, an *i*_d_ variation equal to 0.1% (compared to the steady-state value) was obtained, while the tensile stress caused a 0.4% variation. Overall, the observed *i*_d_ fluctuations ascribable to mechanical deformations can be considered negligible compared to the current changes induced by UA oxidation.

### Arduino BLE Setup Response

The point-of-care analysis approach, when considering the medical field of applications, implies not only the presence of a compact, non-invasive sensor but also the integration of the analytical device with a suitable electronic readout system, which ideally should be low cost and user friendly. For this reason, a proof-of-concept test was performed using an Arduino BLE-based instrumental setup connected via Bluetooth to a smartphone app, with real-time display and data logging capabilities, as illustrated in Figure 7. Upon addition of microliter volumes of UA on the surface of the sensor, a sharp decrease in *i*_d_ can be seen on the screen. By sampling the current on the maximum recorded value and normalizing the data to the steady-state current relative to a UA concentration of 100 μM, it was possible to build a calibration plot, whose NCR slope was found to be 0.39 ± 0.02 decade^–1^. If the confidence interval is associated with the average slope reported for the OECTs tested using the bipotentiostat (*N* = 14), we obtain 0.52 ± 0.15 decade^–1^. Therefore, the value reported for the slope obtained by Arduino setup is part of the global NCR slope distribution containing 95% of the devices. The result of this test confirms that it is possible to miniaturize the electronics necessary to supply an OECT and record the relevant electrical parameters, allowing for a significant cost and hindrance reduction without affecting the sensor performance.

### Comparison with Other State-of-the-Art Sensors for Uric Acid Detection

The sensing performance of the smart dressing developed in this work was compared with some recent electrochemical devices for uric acid monitoring. Table 2 features the most relevant sensing parameters. Considering the average UA biological concentrations in wound exudate^41^ (220–750 μM), our device displays a response range and a limit of detection (LOD) more than sufficient for biomedically relevant quantification. Such performances are overall better than most textile-based devices found in the scientific literature, as they appear to have a more limited range of responses. Regarding the sensors’ architecture, our device is, to the best of our knowledge, the first fully textile OECT to have been tested in flow conditions using synthetic wound exudate for UA determination. Moreover, the choice of the referenceless transistor architecture, the simple potentiostatic technique, together with the low operating voltages, and the amplified output signal make the smart dressing highly compatible with low-cost, portable electronic readouts for future on-field application of this technology, as demonstrated by the development of the Arduino-based reading platform. On the other side, our devices display the highest response time, which can be ascribed to the intrinsic analysis conditions (i.e., low flow injection rate to better simulate the wound fluid excretion) and to the high electrochemically active surface area of the device. It is also worth noting that, although enzymatic transduction is one of the most popular choices for UA sensing, the smart dressing simply relies on the direct UA oxidation on the printed semiconducting ink, thus positively impacting the device robustness and final cost and still guaranteeing the selectivity required for investigating the wound environment.

**Table 2:** Analytical Performance Comparison for Various Types of Uric Acid Sensors^a^

setup	transduction	response range	response time	LOD	textile based	analysis mode	matrix	ref
OECT (potentiostatic)	direct oxidation on PEDOT:PSS	110–1100 μM	439 s	75 μM	YES	progressive additions in electrochemical cell	PBS	this work
OECT (potentiostatic)	direct oxidation on PEDOT:PSS	200–1000 μM	n/a	76 μM	YES	discrete additions on the sensor, arduino powered	PBS	this work
OECT (potentiostatic)	direct oxidation on PEDOT:PSS	100–1000 μM	702 s	23 μM	YES	flow conditions analysis	SWE	this work
3-electrode (CA)	UOx-BSA on Prussian blue	100–800 μM	60 s	n/a	YES	discrete additions on the sensors	PBS	Kassal 2015^47^
3-electrode (CA)	UOx on carbon ink	0–800 μM	60 s	n/a	YES	discrete additions on the sensors	SWE	Liu 2017^48^
3-electrode (DPV)	UOx-PVA-SbQ on carbon ink	0–300 μM	45 s	n/a	YES	discrete additions on the sensors	human wound exudate	RoyChoudhury 2018^49^
3-electrode (DPV)	UOx-BSA on LGG-MXene	50–1200 μM	n/a	50 μM	NO	discrete additions on the sensors	SWE	Sharifuzzaman 2020^38^
3-electrode (CV)	AuNPs on CPE	0–500 μM	50 s	n/a	YES	immersion in electrochemical cell	PBS	Wu 2023^87^
3-electrode (potentiostatic)	CuWO_4_ NPs on SPCE	0.001–298.2 μM	n/a	0.2 nM	NO	immersion in electrochemical cell	BSA/human urine	Sriram 2023^88^

a CA: chronoamperometry; UOx: urate oxidase; BSA: bovine serum albumin; DPV: differential pulse voltammetry; PVA-SbQ: poly(vinyl alcohol) *N*-methyl-4(4′-formylstyryl)-pyridinium-metho-sulfate-acetal; LGG: laser-guided graphene; NPs: nanoparticles; CPE: carbon-paste electrode; and SPCE: screen-printed carbon electrode.

### In Vitro Cytotoxicity Trials

In view of the future on-skin target application of the smart dressing for UA monitoring, an in vitro cytotoxicity study was carried out by an accredited laboratory following the ISO 10993-5:2009 method, as detailed in the Experimental Section. In vitro cytotoxicity is a primary biocompatibility test result, and it was carried out by exposing a mammal fibroblasts cell line to the sensing layer of the smart dressing, i.e., the printed layer with the OECT sensor. Qualitative and quantitative assessments were performed through microscopic observation and optical density evaluation of the cell’s viability, respectively. In the first case, biological reactions in the different wells were evaluated after incubation time following a 0 (none) to 4 (severe reactivity) scale. No reactivity zones were detected in proximity to the test sample. Concurrently, quantitative analysis revealed a cell viability reduction of only 2.0 ± 0.8% among the cells exposed to the test sample. Therefore, the sensing layer of the smart dressing was certified as not cytotoxic.

## Conclusions

Nowadays, few to no commercial smart dressings are able to monitor the wound-healing status without removing the bandages and visually inspecting the affected area. Since real-time monitoring of UA could be a powerful diagnostic tool to gain valuable information on wound-healing stages and thus the well-being of the patient, recent literature has proposed some examples of smart dressings for UA monitoring based on enzymatic transduction. However, these devices are far from commercialization due to the increased manufacturing cost and the need for delicate handling conditions associated with the use of an expensive and fragile enzyme. In this project, a smart dressing based on an OECT has been designed and developed with an innovative cross-like geometry for selective and enzyme-free UA monitoring wherein the gate electrode is glued onto the channel. After optimizing the geometry and developing a sampling system based on medical-grade materials, the sensor performances have been assessed in both static and flow conditions, showing that the device can operate in an artificial exudate within a UA concentration range relevant for medical applications. The sensor does not exhibit a response to the most common interferents present in the wound exudate, thus highlighting its high selectivity. Moreover, an Arduino board has been employed to recover relevant data demonstrating the possibility of sensor management with low-cost readout electronics. In conclusion, a fully textile-smart dressing for the determination of uric acid in synthetic wound exudate was successfully developed and optimized, displaying good repeatability, reproducibility, and selectivity. The non-enzymatic approach grants lower manufacturing costs and easier storage conditions, while the OECT architecture gives a significant advantage over the conventional three-electrode cell-based systems since it does not require a reference electrode and it allows for intrinsic signal filtering and amplification. The presented results are a springboard for increasing the technological maturity level of smart dressing filling the gap between research and commercialization.

## Supporting Information Available

SEM images of A030THI, Jettex 1005, and PHT 3093 Foam; pictures of the printing resolution obtainable using Jettex 1005 and Royal 100; transfer and output curves relative to each textile OECT geometry; glass-based OECT *V*_gs_ optimization; *E* (vs SCE) vs *V*_gs_ experiments on glass-based OECTs; pH influence on UA oxidation peak in cyclic voltammetry; *i*_d_ steady state in flow conditions obtained in PBS and SWE; textile OECT response in flow conditions by random UA additions; repeatability test upon flow delivery of a fixed UA concentration on successive days; and image of the setup used to perform mechanical deformation test (PDF)
