# Supplementary material for: Smart Bandaid Integrated with Fully Textile OECT for Uric Acid Real-Time Monitoring in Wound Exudate
Source: ACS Sens. 2023 Mar 17;8(4):1593–608. doi: 10.1021/acssensors.2c02728 (PMC10152490; doi:10.1021/acssensors.2c02728)
Supplement: Supplementary file 1 — se2c02728_si_001.pdf [file se2c02728_si_001.pdf]

Supporting Information

Smart bandaid integrated with fully-textile OECT for Uric Acid Real-Time Monitoring in Wound Exudate

Danilo Arcangeli[1], Isacco Gualandi[1], Federica Mariani[1,*], Marta Tessarolo[2], Francesca Ceccardi[1], Francesco Decataldo[2], Federico Melandri[3], Domenica Tonelli[1], Beatrice Fraboni[2], Erika Scavetta[1]

[1] Department of Industrial Chemistry "Toso Montanari", University of Bologna, Viale Risorgimento 4, 40136, Bologna, Italy

[2] Department of Physics and Astronomy "Augusto Righi", University of Bologna, Viale Berti Pichat 6/2, Bologna, 40127, Italy

[3] Plastod S.p.A., Via Walter Masetti 7, 40012, Calderara di Reno, Bologna, Italy

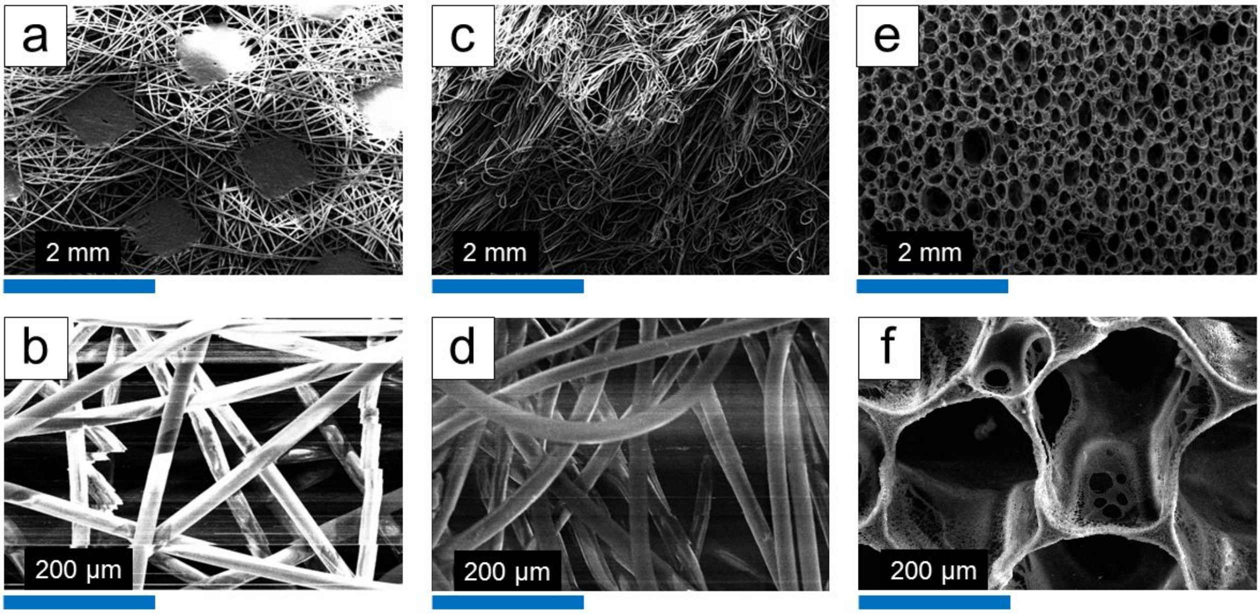

Figure S1 – SEM imaging of the textile materials (**a; b**) A030THI, (**c; d**) Jettex 1005 and (**e; f**) PHT 3093 foam.

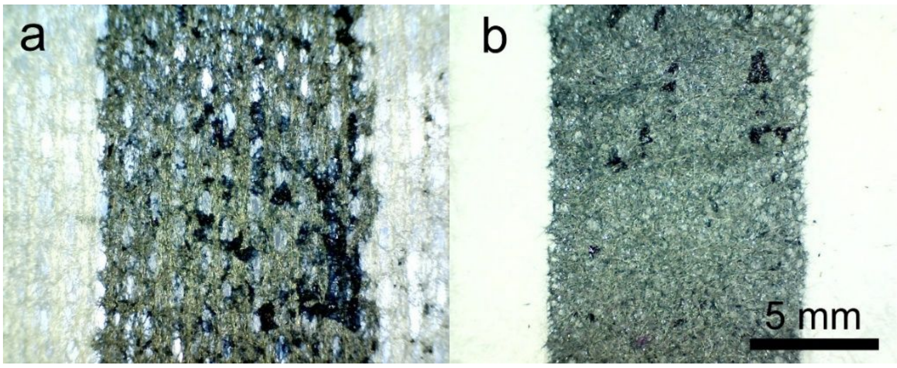

Figure S2 – Digital microscope images of PEDOT:PSS-based ink screen printed on (**a**) Jettex 1005 and (**b**) Royal 100 gauzes.

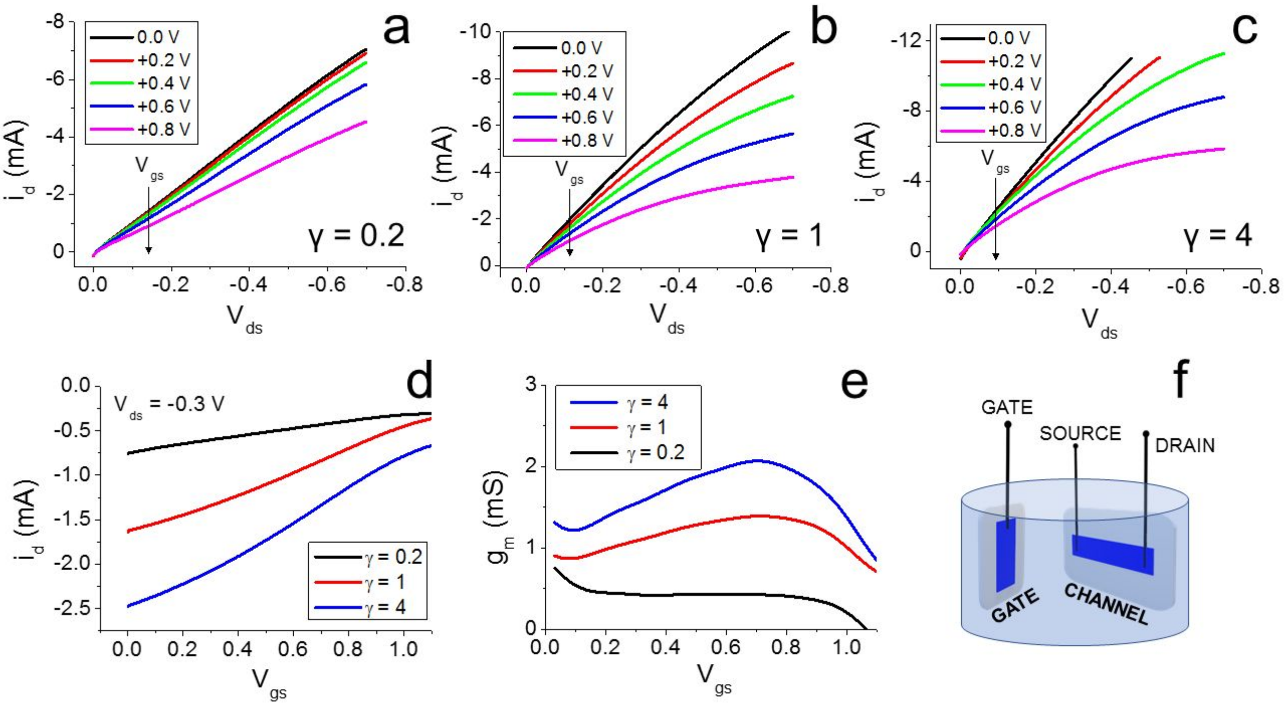

Figure S3 – Output curves recorded for a textile OECT in 0.1 M PBS electrolyte (pH = 7.00) with (**a**) γ = 0.2; (**b**) γ = 1; (**c**) γ = 4. (**d**) Transfer curves obtained in the same conditions for each geometry tested and (**e**) their relative transconductance curves. (**f**) Representation of the electrochemical cell setup used to record transfer and output curves.

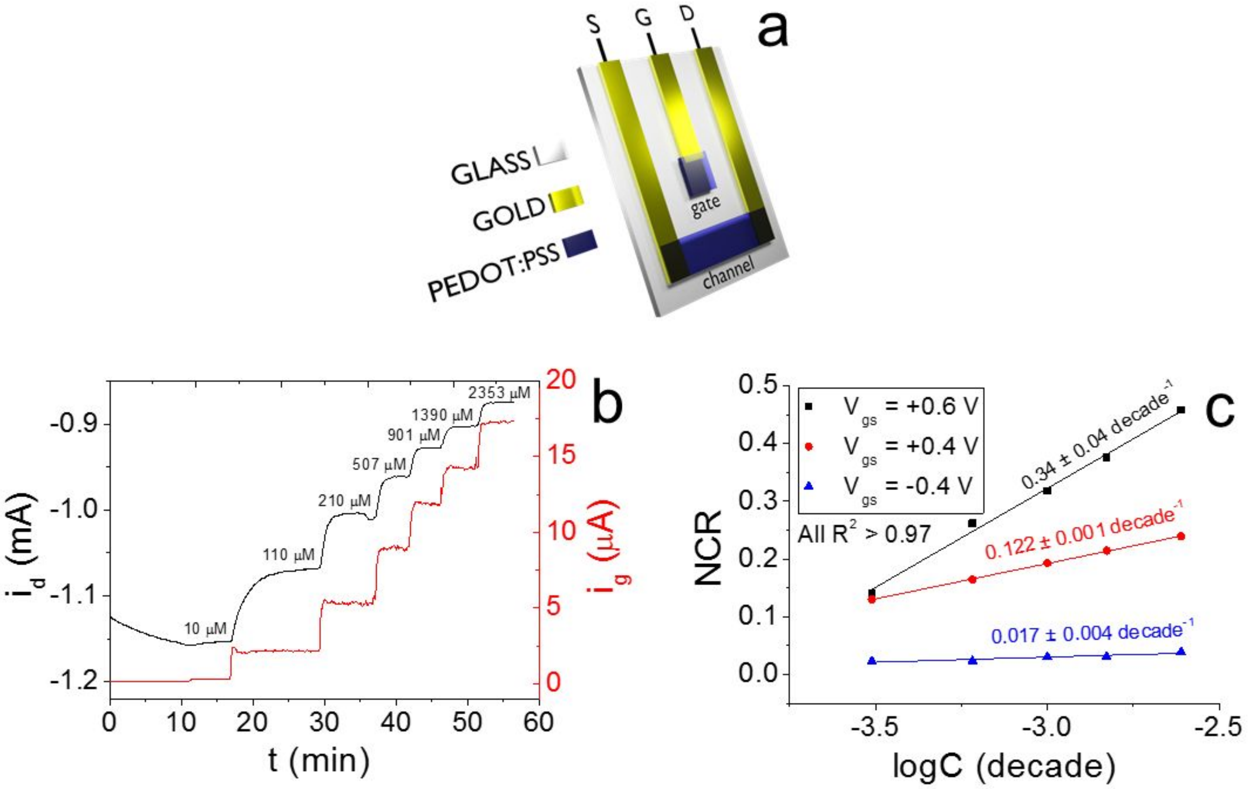

Figure S4 – (**a**) Glass based thin-film PEDOT:PSS OECT and (**b**) its response to UA additions in 0.1 M PBS electrolyte (pH = 7.00). (**c**) Effect of V_{gs} on the NCR slope value.

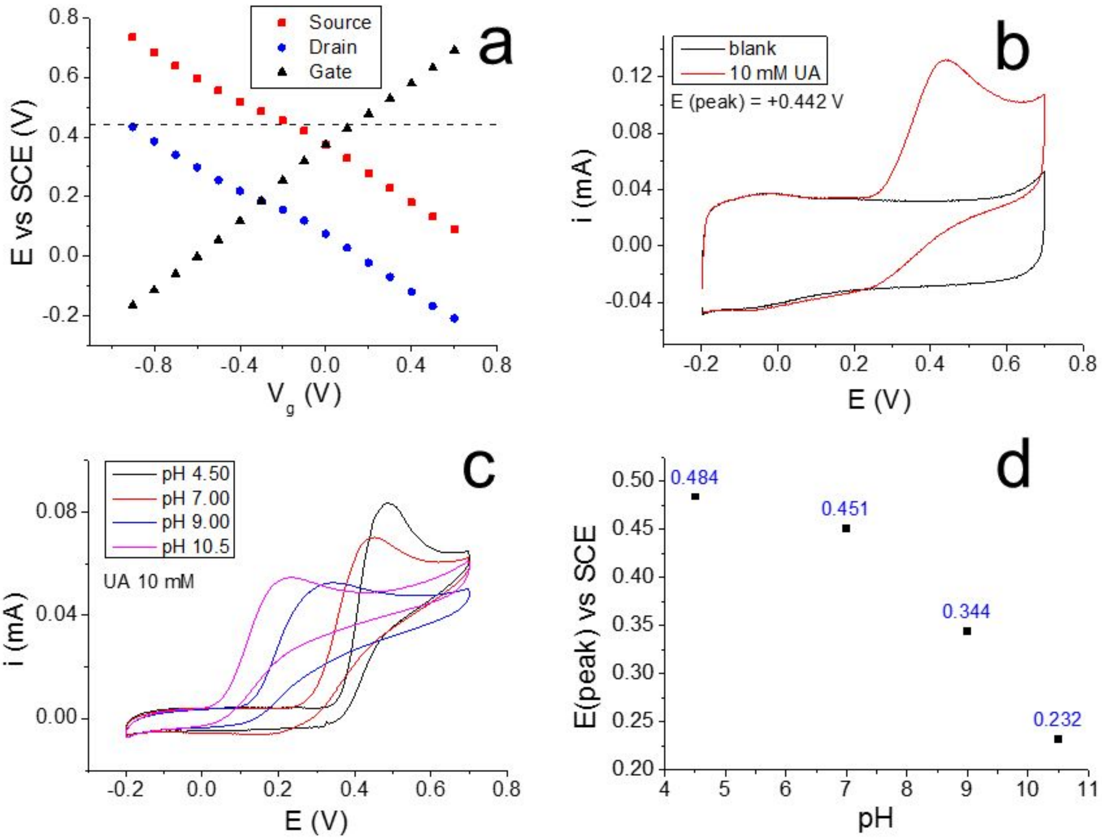

Figure S5 – (**a**) Electrochemical potential measurements vs SCE of a glass-based OECT terminals by variation of gate voltage, UA oxidation peak voltage is reported as horizontal dashed line. (**b**) Cyclic voltammetry response of UA on a PEDOT:PSS film supported on glass in PBS 0.1 M @ pH 7.00 vs SCE. (**c**) Effect of the pH in cyclic voltammetry response of UA in Universal Buffer. (**d**) UA peak potential vs pH.

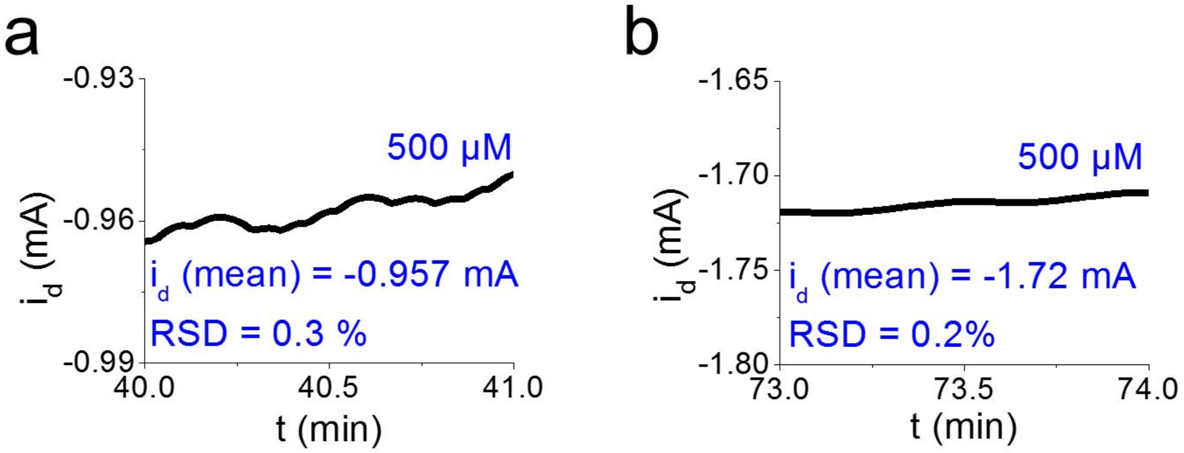

Figure S6 – (**a**) Zoomed steady-state current reached during flow conditions testing at UA = 500 µM in PBS. (**b**) Zoomed steady-state current reached during flow conditions testing at UA = 500 µM in SWE.

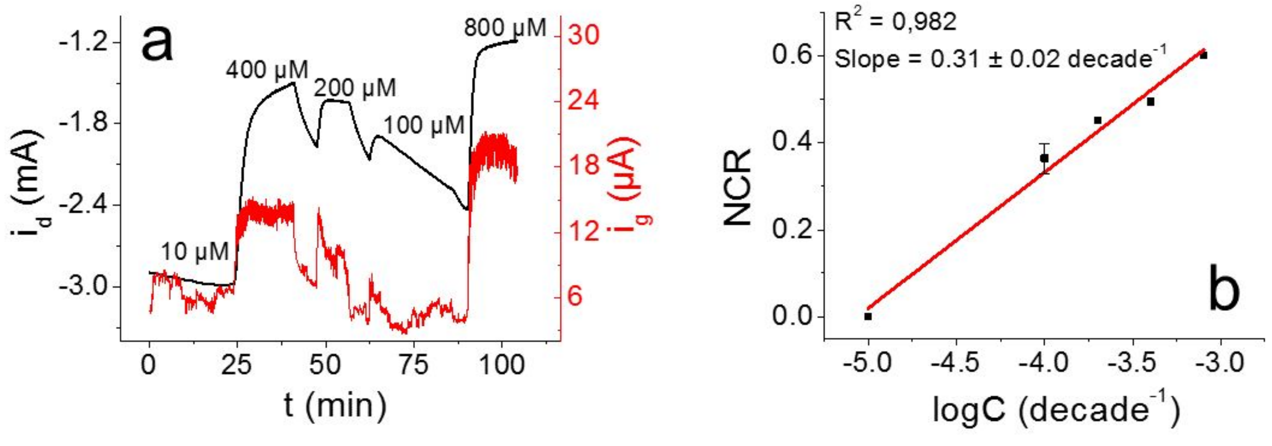

Figure S7 – (**a**) Flow conditions analysis response on a textile OECT by random addition of UA solutions in PBS, V_{ds} = -0.3 V; V_{gs} = +0.6 V and (**b**) its relative calibration plot.

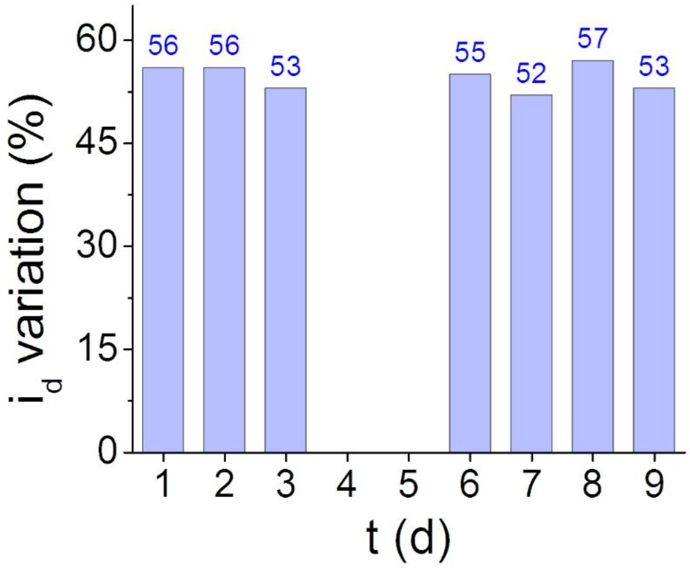

Figure S8 – Stability test performed in flow conditions for an assembled textile OECT upon delivery of a 200 µM UA solution in 0.1 M PBS at pH 7.00. V_{ds} = -0.3 V; V_{gs} = +0.6 V; flow rate = 0.05 mL/min.

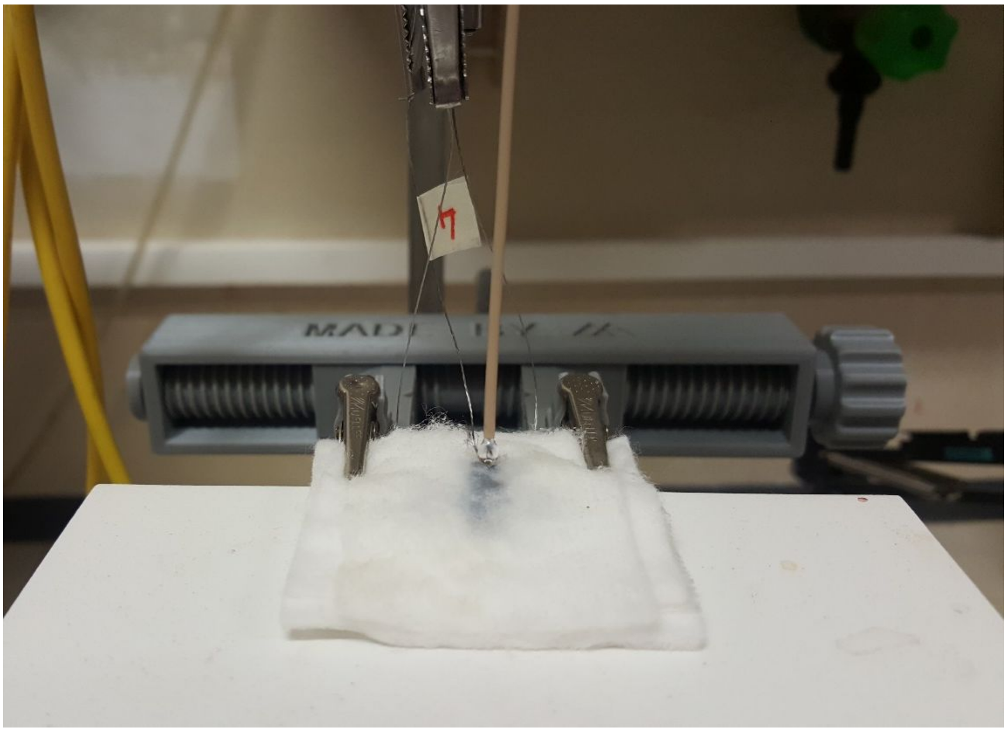

Figure S9 – Fully-assembled textile OECT attached to the custom-made 3D printed vise for mechanical deformation testing.
